# Icaritin eliminates tumor-associated macrophages via STX16-dependent extracellular vesicle delivery of autophagosomes from hepatocellular carcinoma cells

**DOI:** 10.1186/s13046-026-03671-0

**Published:** 2026-02-23

**Authors:** Xia Zheng, Wenshu Qu, Chen Xun, Chao Zhang, Yuan Li, Xinyu Xu, Yang Gao, Yu Gu, Zhihui Yang, Xing Huang, Jun Qian

**Affiliations:** 1 Department of Oncology, Affiliated Hospital of Nanjing University of Chinese Medicine, Nanjing, China; 2 Department of Oncology, Nanjing Tianyinshan Hospital of China Pharmaceutical University, Nanjing, China; 3 Basic Medical Research Innovation Center for Anti-Cancer Drugs, China Pharmaceutical University, Nanjing, China; 4 Department of Pathology, Affiliated Hospital of Nanjing University of Chinese Medicine, Nanjing, China; 5 Department of Pathology, Jiangsu Cancer Hospital, Nanjing, China; 6 Department of Radiology, Jiangsu Cancer Hospital, Nanjing, China; 7 Department of Pathology, Nanjing Jinling Hospital, Nanjing, China; 8 Department of General Surgery, Affiliated Hospital of Nanjing University of Chinese Medicine, Nanjing, China

**Keywords:** Hepatocellular carcinoma, Icaritin, Histone lactylation, Autophagolysosome biogenesis, Tumor-associated macrophage

## Abstract

**Background:**

To elucidate the mechanism by which icaritin—a novel agent for hepatocellular carcinoma (HCC)—remodels the tumor microenvironment (TME) by inhibiting HCC cell metabolism-mediated M2 polarization of tumor-associated macrophages (TAMs).

**Methods:**

Integrative approaches spanning in vitro Transwell cocultures, RNA-seq, LC3-based autophagy tracing, STX16 gene edition, and orthotopic xenografts mechanistically dissected the affective and mechanism of icaritin in remodeling TME.

**Results:**

Our results indicate that icaritin transcriptionally suppresses ALDOB in HCC cells to reduce lactate production. Consequently, the lactylation of histone H3 at lysine 9 and lysine 18 (H3K9/H3K18la) on the STX16 promoter is diminished, thereby ablating STX16 transcription. STX16 deficiency blocks autophagolysosome biogenesis, leading to the accumulation of autophagosomes in HCC cells. These autophagosomes are subsequently delivered to macrophages via extracellular vesicle (EVs) and then triggers autophagic cell death and p62-guided STAT3 destruction within the macrophages, thereby eliminating M2 TAMs and reprograming the tumor immune microenvironment. In vivo validation confirmed icaritin suppressed tumor growth and M2 macrophage infiltration via the ALDOB/STX16/autophagy/STAT3 axis.

**Conclusion:**

These results indicate that by orchestrating a novel "metabolism-epigenetics-autophagy-EVs" cascade to eliminate TAMs, icaritin targets ALDOB, STX16, and STAT3, revealing key nodes for therapeutic intervention.

**Graphical Abstract:**

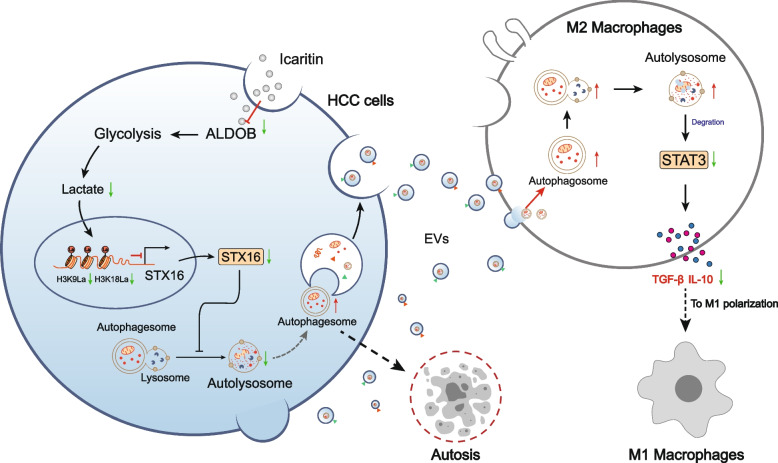

**Supplementary Information:**

The online version contains supplementary material available at 10.1186/s13046-026-03671-0.

## Introduction

As the most prevalent pathological subtype of primary liver cancer, hepatocellular carcinoma (HCC) ranks fifth in incidence and third in mortality of cancer globally [1]. In China, it represents the fourth common malignancy and the second leading cause of cancer-related death [2]. Due to lack of specific symptoms, most HCC patients developed to advanced disease with metastasis when initial diagnosis, missing the opportunity to receive radical treatment options such as hepatectomy [3]. Historically, tyrosine kinase inhibitors (TKIs) such as sorafenib and lenvatinib were limited by modest objective response rates (ORR) of 3–18% and the frequent development of acquired resistance within six months. Subsequently, immune checkpoint inhibitor (ICIs) monotherapy demonstrated improved responses (ORR: 15–20%), yet primary resistance affected more than 80% of patients [4]. Despite the breakthrough of modern combinations like ICIs with anti-angiogenics, their efficacy remains constrained by a modest objective response rate (~ 30%), unsatisfactory overall survival, and a 5-year survival rate below 20% [5–7]. Consequently, developing novel therapies that can extend survival has sparked considerable interest in the field of advanced HCC.

Given the absence of well-defined oncogenic drivers in HCC, therapeutic strategies have predominantly focused on modulating tumor microenvironment (TME). Macrophages, recognized as pivotal cellular constituents of the TME, exhibit marked heterogeneity and plasticity [8]. Generally, M1 macrophages functioned as stimulating Th1-type immune responses, activating effector T cells, and promote anti-tumor immune reactions. In contrast, M2 macrophages were identified as an immune suppression and tumor promotion sub-population by secreting interleukin 10 (IL-10) and transforming growth factor beta 1 (TGFB1) [9, 10]. Tumor-associated macrophages (TAMs) exhibit functional and phenotypic similarities to M2 macrophages, thereby significantly contributing to tumor progression, immune evasion, and resistance to immunotherapy. Accumulating evidence from previous investigations has established that bidirectional material exchange and intercellular signaling transduction existed between HCC cells and macrophages, which collectively promoted M2 polarization of macrophages and mediated immune-suppressive in TME [11–13]. Strategies to reprogram M2 macrophages toward M1 phenotype to re-activate immune-inflammatory responses, or to inhibit TAMs recruiting and infiltrating, represent promising novel approaches in cancer therapy including HCC [14–16]. However, no drugs with the function of shifting macrophage polarization have approved for cancer treatment so far.

Supported by clinical evidence, the small-molecule agent icaritin is now an approved first-line treatment option for advanced HCC in China [17, 18]. The anticancer activities of icaritin mainly associated to regulate IL-6/JAK/STAT3 pathways in tumor cells and immune cells including CD8 + T cells [19], myeloid-derived suppressor cells [20], neutrophils, and macrophages [17]. Additionally, our previous study revealed that icaritin inhibited GLUT1-mediated glycolysis in HCC cells via FAM99A/JAK2/STAT3 pathway [21]. Interestingly, it has been revealed that targeting HCC cells glycolysis could reprogram macrophages polarization in TME [22, 23]. Taken these considerations, we hypothesize that icaritin might exhibit the effect of remodeling macrophage polarization through regulating HCC cells glycolysis. In current study, we demonstrated that icaritin depleted HCC cells-educated M2 polarization macrophage by EVs-transported autophagosomes. Within these complex biological processes, ALDOB-mediated glycolytic reprogramming and STX16-driven autophagolysosomes biogenesis were demonstrated to execute indispensable roles.

## Methods

### Cell culture

Human immortalized hepatocytes THLE2, human HCC cell lines HepG2, Hep3B, and monocytic THP-1 were obtained from the American Type Culture Collection (ATCC). THLE2 cells are typically cultured in DMEM/F-12 medium with 10% FBS, EGF (20 ng/mL), insulin (5 μg/mL), transferrin (5 μg/mL), sodium selenite (5 ng/mL), and hydrocortisone (0.1 μmol/L). HepG2 and Hep3B cells were maintained in DMEM (Procell, PM153210) supplemented with 10% fetal bovine serum (FBS) and 1% penicillin/streptomycin (Gibco, 1,514,022). THP-1 cells were cultured in RPMI-1640 (Procell, PM153110) with 10% FBS and 1% penicillin/streptomycin. All cells were incubated at 37 °C in a 5% CO₂ humidified atmosphere.

THP-1 monocytes were differentiated into M0 macrophages by treatment with 100 ng/mL phorbol 12-myristate 13-acetate (PMA) (Biosharp, BL1127A) for 24 h. Cells were then washed thrice with PBS and cultured in complete RPMI-1640 medium for an additional 24 h prior to experiments.

### Co-culture system establishment

**Fig. 1:**
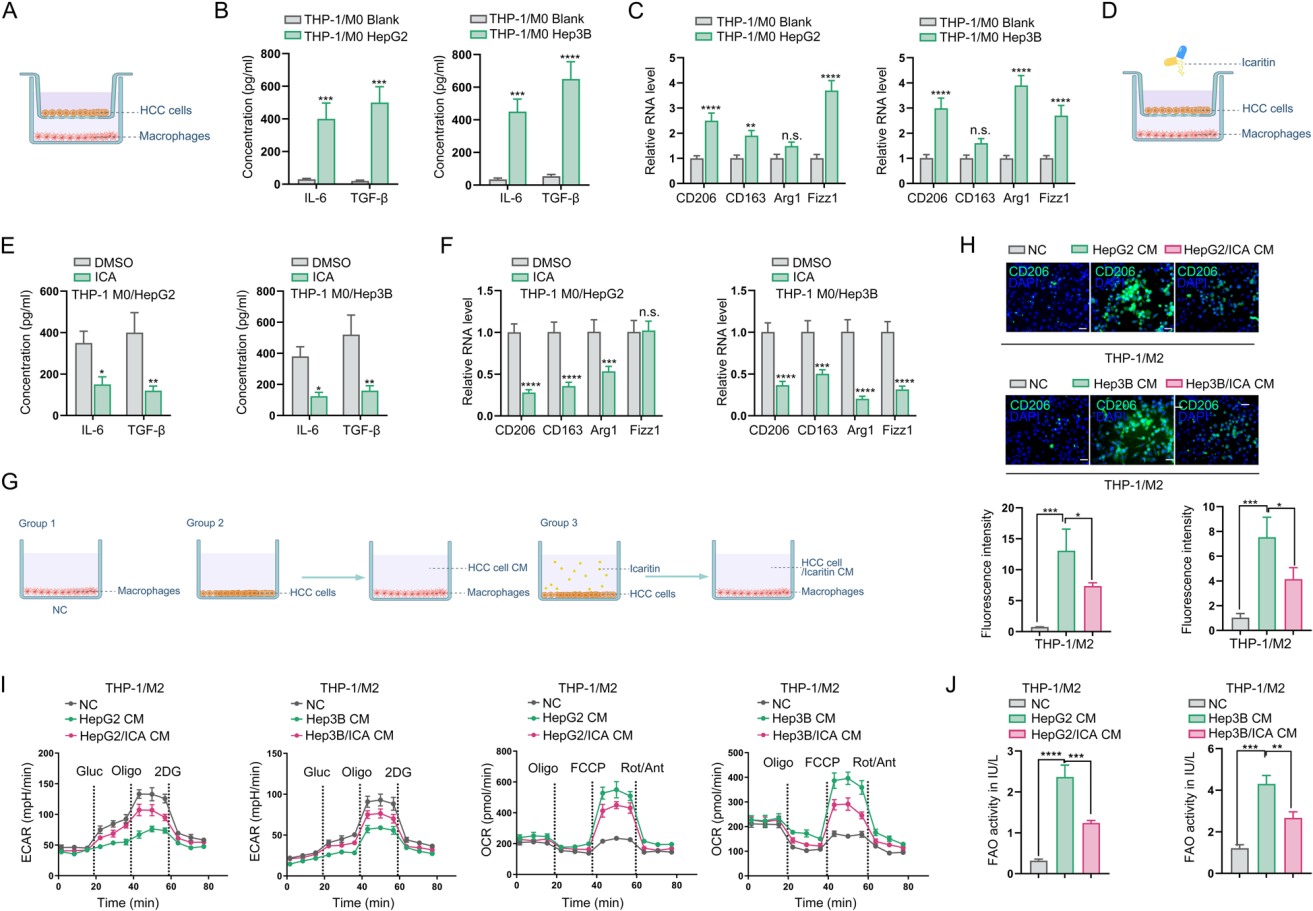
Icaritin diminished HCC cell-mediated M2 polarization of macrophages by reprogramming metabolism. **A** Schematic of HCC-macrophage co-culture in vitro model establishment; **B** Detection of IL-6 and TGF-β secretion via ELISA; **C** Examination of M2 polarization markers by qPCR; **D** Schematic diagram of the HCC-macrophage coculture system administrated using icaritin in vitro; **E** ELISA quantification of IL-6 and TGF-β secretion following icaritin administration; **F** Detection of M2 polarization markers in macrophages after icaritin administration; **G** Schematic of macrophages administrated with the CM from HCC cells pretreated with or without icaritin. **H** IF staining of CD206 expression in macrophages; **I** ECAR and OCR analysis of glycolysis in macrophages; **J** Assessment of FAO activity in macrophages treated with CM from icaritin- or DMSO-exposed HCC cells (*P < 0.05, **P < 0.01, ***P < 0.001, ****P < 0.0001, n.s.: no significance)

We established a Transwell co-culture system using 24-well plates with 0.4-μm pore polycarbonate membrane inserts (Fig. 1A). THP-1 monocytes (1 × 10^5^ cells/mL) were seeded in the lower chamber and differentiated into M0 macrophages by treatment with 100 ng/mL PMA for 24 h, followed by three PBS washes and 24 h culture in complete RPMI-1640 medium. Subsequently, HepG2 or Hep3B cells (1 × 10^5^ cells/mL) were plated in the upper chamber and allowed to adhere for 6 h before initiating 24 h co-culture with the differentiated macrophages. Following co-culture, conditioned media (CM) were collected and sequentially centrifuged at 300 × g for 10 min and 2,000 × g for 10 min (4 °C) to remove cells and debris, then filtered through 0.22-μm membranes for subsequent functional assays (Fig. 1G).

### Transfection

Specific siRNAs targeting STX16 (si-STX16) and SQSTM1/p62 (si-p62), along with negative control siRNA (si-NC), were designed and synthesized by GenePharma. For overexpression assays, the recombinant plasmid pcDNA3.1-STX16 (YouBio, H5723) and empty vector pcDNA3.1 (Invitrogen, V79020) were utilized. Cells were seeded in 6-well plates until reaching 60–70% confluency prior to transfection. Plasmid DNA or siRNA (2.5 μg/well) was mixed with Lipofectamine™ 3000 Reagent (Invitrogen, L3000008) in serum-free Opti-MEM medium (Gibco, 31,985,070), incubated for 20 min at room temperature to form transfection complexes, and added dropwise to cell cultures. After gentle swirling to ensure uniform distribution, cells were incubated for 48–72 h at 37 °C before harvest. Transfection efficiency was validated via qPCR and Western blot. The primers sequences of transfected gene are listed in Table S1.

### RNA extraction and quantitative real-time PCR (RT-qPCR)

Total RNA was extracted from tissue and cell samples using TRIzol Reagent (Invitrogen, 15,596,026). RNA concentration and purity were determined by absorbance measurement at 260/280 nm using a NanoDrop One spectrophotometer (Thermo Fisher Scientific, ND-ONEC-W). First-strand cDNA synthesis was performed with 1 μg total RNA using the HiScript III All-in-One RT SuperMix Perfect for qPCR kit (Vazyme, R333) according to manufacturer's protocol. RT-qPCR amplification was carried out using Taq Pro Universal SYBR qPCR Master Mix (Vazyme, Q712) on a QuantStudio 6 Flex Real-Time PCR System (Applied Biosystems) under the following cycling conditions: 95 °C for 30 s; 40 cycles of 95 °C for 10 s and 60 °C for 30 s. ACTB (β-actin) served as the endogenous reference gene. Relative gene expression was calculated using the 2^−ΔΔCt^ method. Primer sequences are listed in Table S2.

### Enzyme-Linked Immunosorbent Assay (ELISA) analysis

Cytokine levels (IL-6 and TGF-β1) in CM were quantified using commercial ELISA kits (Beyotime PI330 for IL-6, PT880 for TGF-β1) following manufacturer protocols. Briefly, samples were centrifuged (10,000×g, 10 min), diluted 1:5, and incubated in antibody-coated wells (2 hr, 37 °C). After washing, detection antibodies (1h, 37 °C) and streptavidin-HRP (30min, 37 °C) were sequentially added, followed by TMB substrate development (15min) and reaction termination with 2 N H₂SO₄. Absorbance was measured at 450/570 nm, with concentrations calculated against standard curves (0–500 pg/mL). All samples were analyzed in triplicate (CV<10%).

### Metabolic flux analysis

Cellular bioenergetics were analyzed using a Seahorse XFe24 Analyzer. For mitochondrial stress tests, 5 × 10^4^ cells/well were assayed in XF DMEM medium (Seahorse, 103,575–100) with sequential injection of 1 μM oligomycin (ATP synthase inhibitor), 1.5 μM FCCP (mitochondrial uncoupler), and 100 nM rotenone/1 μM antimycin A (ETC inhibitors), along with 25 μM BPTES (glutaminase inhibitor) and 7.5 mM MSO (glutamine synthetase inhibitor). Glycolytic function was assessed via ECAR measurements following 10 mM glucose, 1 μM oligomycin, and 20 mM 2-DG (glycolysis inhibitor) challenges. Data were normalized to protein content and analyzed using Wave Software 2.6.

### Fatty Acid Oxidation (FAO) assay

FAO activity was quantified using a Human FAO ELISA Kit (COIBO BIO, CB13660-Hu) according to the manufacturer's instructions. Briefly, samples and standards were added to antibody-precoated wells, incubated with HRP-conjugated detection antibody (1 h, 37 °C), washed, and developed with TMB substrate (15 min). The reaction was stopped with 2 N H₂SO₄, and absorbance was measured at 450 nm (reference: 630 nm). FAO concentrations were calculated from a standard curve (0–50 ng/mL) with triplicate measurements (CV < 8%).

### Cell viability assay (CCK-8)

Cell viability was assessed using CCK-8 reagent (Beyotime, C0038). For HCC cells, STX16-overexpressing or control cells treated with icaritin (0–8 μM) were seeded in 96-well plates (5 × 10^3^ cells/well), incubated with 10 μL CCK-8 for 2 h at 37 °C, and measured at OD450. For macrophages, cells (1 × 10^4^ cells/well) treated with HCC-derived EVs were monitored daily at OD450 for 72 h to generate growth curves. Viability was calculated as: (OD_treated_-OD_blank_)/(OD_control_-OD_blank_) × 100%.

### Western blot analysis

Protein samples were extracted using RIPA lysis buffer (Beyotime, P0013B) and quantified with BCA assay (Beyotime, P0009). Equal amounts (30 μg) of protein were separated by SDS-PAGE and transferred to PVDF membranes. After blocking, membranes were incubated with primary antibodies (Table S3) overnight at 4 °C, followed by HRP-conjugated secondary antibodies (1:5000) for 1 h at room temperature. Protein bands were visualized using BeyoECL Star chemiluminescent substrate (Beyotime, P0018AM) and imaged with a ChemiDoc system.

### Immunofluorescence (IF) staining

Cells were fixed with 4% PFA (5 min), washed with PBS (3 ×), and blocked for 2 h at RT, while tissues were fixed in 4% PFA (24 h), paraffin-embedded, sectioned (5 μm), and similarly processed. Samples were incubated with primary antibodies (Table S3) overnight at 4 °C, followed by fluorophore-conjugated secondary antibodies (1:500, 1 h, RT) and DAPI nuclear counterstain (Beyotime, C1002). Images were acquired using a Zeiss LSM800 confocal microscope.

### Lactate secretion assay

Lactate levels were quantified using the Lactate Assay Kit II (Sigma-Aldrich, MAK065) following the manufacturer's protocol. Briefly, 25 µL of samples were diluted 1:2 in lactate assay buffer in a clear 96-well plate, mixed with 50 µL reaction reagent per well, and incubated for 30 min at RT in the dark. Absorbance (OD450) was measured using an Epoch microplate reader (BioTek), with lactate concentrations calculated against a standard curve.

### Cycloheximide (CHX) chase assay

Cells were seeded in 6-well plates until reaching 80% confluency and synchronized with 10 μM MG132 (MCE, HY-13259). For protein degradation analysis, cells were treated with 100 μg/mL CHX (MCE, HY-12320) and harvested at specified timepoints (0, 3, 6, and 9 h). In parallel, TAMs-M2 pretreated with HCCs/ICA EVs were exposed to proteasomal (MG132) or autophagic inhibitors (3-MA [5 mM], CQ [20 μM], BafA1 [100 nM]; all from MCE) before CHX treatment. Cell lysates were analyzed by Western blot to assess STAT3 protein stability.

### Propidium Iodide (PI) flow cytometry assay

Cells were washed twice with PBS and centrifuged (1,000 × g, 5 min). The pellet was resuspended and stained with PI solution (Yeasen, 40711ES10) for 1 h at RT in the dark. Samples were analyzed using a CytoFLEX flow cytometer (Beckman Coulter), with ≥ 3 biological replicates performed.

### Intracellular pH measurement

Cells were loaded with 5 μM BCECF-AM (Beyotime, S1006) for 30 min at 37 °C, washed with PBS, and imaged under a fluorescence microscope using 488 nm excitation/535 nm emission filters. The 490/440 nm excitation ratio was calculated for pH quantification, calibrated with pH standard buffers (pH 6.0–8.0) containing 10 μM nigericin.

### Autophagosome-lysosome fusion assay

Cells were transduced with mRFP-GFP-LC3 lentivirus (Hanbio, HB-LP2100001) and selected with puromycin (2 μg/mL, 72 h). Autophagic flux was quantified by confocal microscopy (Zeiss LSM800) based on fluorescence signal conversion.

### Luciferase reporter assay

The STX16 promoter was cloned into pGL3-Basic vector and co-transfected with pRL-TK (internal control) using Lipofectamine 2000 (Invitrogen, 11,668,019). After 48 h, dual-luciferase activity was measured using a detection kit (Vazyme, DL101-01), with firefly luminescence normalized to Renilla values for quantitative analysis of promoter activity.

### ATAC-qPCR analysis

Nuclei were isolated and purified from samples, then resuspended in a Tn5 transposase reaction system and incubated at 37 °C for 30 min to fragment accessible chromatin. After transposition, equimolar amounts of Adapter 1 and Adapter 2 were added for PCR amplification and library construction. Amplified products were purified using a DNA Cleanup Kit (Vazyme, DC301-01) and eluted in 20 μL elution buffer. Chromatin accessibility was analyzed via qPCR using HiScript III All-in-one RT SuperMix Perfect for qPCR (Vazyme, R333).

### ChIP-qPCR analysis

Chromatin immunoprecipitation (ChIP) was performed using the SimpleChIP® Plus Sonication Kit (CST, 56,383). Cells were crosslinked with 1% formaldehyde (10 min), quenched with 0.125 M glycine (5 min, RT), and lysed. Chromatin was sheared to 200–500 bp fragments by sonication. Sheared chromatin was incubated with antibodies overnight at 4 °C, followed by Protein A/G magnetic bead capture and washing with low/high-salt buffers. Immunoprecipitated DNA was eluted, reverse-crosslinked (65 °C), and purified. Enriched DNA was analyzed by qPCR using primers targeting the STX16 promoter: Forward: 5’-CCTCGTGGTTGTTTGTCGGA-3’, Reverse: 5’-GCGGGAGAGGAAAAGTTGGA-3’.

### RNA stability assay (actinomycin D treatment)

To assess RNA degradation kinetics, cells were seeded in 6-well plates and treated with icaritin-induced HCC cells EVs until reaching 70–80% confluency. Transcription was halted by adding 5 μg/mL actinomycin D (ActD; MCE, HY-17559), and cells were harvested at 0, 3, 6, and 9 h post-treatment. Total RNA was isolated using TRIzol, reverse-transcribed into cDNA, and analyzed by qPCR. RNA decay rates were calculated using the 2^−ΔΔCt^ method, with degradation curves plotted (time vs. relative mRNA expression) and RNA half-life (t₁/₂) determined via nonlinear regression in GraphPad Prism 10.

### Preparation and identification of EVs

EVs were isolated from CM by ultracentrifugation. The specific steps are as follows: first, dead cells were removed by centrifugation at 300 × g for 10 min, then cell debris was removed by sequential centrifugation at 2,000 × g for 10 min and 10,000 × g for 30 min. Subsequently, the supernatant was ultracentrifuged at 100,000 × g for 70 min to obtain EVs microvesicles as precipitates. The precipitate was washed with phosphate-buffered saline and then ultracentrifuged again with the same parameters. Finally, the precipitate was resuspended in RIPA lysis buffer (Beyotime, P0013B) and incubated on ice for 3 min to prepare EVs lysate. All centrifugation steps were performed at 4 °C. The extracted EVs were identified by electron microscopy, Nanoparticle tracking analysis (NTA), and Western blot.

### Nanoparticle Tracking Analysis (NTA)

A 100 nm standard microsphere (3100A, ThermoFisher) was diluted 250,000-fold with ultrapure water. 1 mL of the diluted solution was used for automatic calibration of the Nanoparticle Tracking Analyzer (Zetaview-PMX120-Z, Particle Metrix). After calibration, the sample cell was washed with 1 × PBS. The EVs sample to be tested was diluted to an appropriate concentration with PBS and then loaded for detection. On the software interface, the sample dilution factor was entered. After confirming that the particle counts at each detection site were similar, "Measurement" and "Run Video Acquisition" were clicked sequentially. The sample name, save path were set, and the corresponding SOP was selected to start the test. The instrument automatically completed the detection and analysis and generated a test report containing information such as particle size and concentration.

### EdU (5-Ethynyl-2'-deoxyuridine) assay

EdU staining was performed using the BeyoClick™ EdU Cell Proliferation Assay Kit (Beyotime, C0075S) strictly following the manufacturer's instructions. Cells were seeded in 12-well plates containing cell climbing slices. After completion of culture, EdU reagent was added to each well, and the cells were incubated at 37 °C for 4 h. The cell climbing slices were washed with PBS and fixed with 4% paraformaldehyde. Subsequently, the slices were permeabilized with 0.3% Triton X-100 in sequence, followed by incubation with Click reaction solution for staining. Finally, the cell nuclei were counterstained with DAPI.

### Flow cytometric detection of Reactive Oxygen Species (ROS)

The Reactive Oxygen Species Detection Kit (Beyotime, S0035S) was used for detection, and the operation was performed according to the kit instructions. 1 × 10^4^ cells were collected for each sample. DCFH-DA was added to the cell culture medium, and the cells were incubated at 37 °C in the dark for 30 min. After staining, the cells were washed with PBS and detected using a flow cytometer (CytoFLEX).

### Immunoprecipitation (IP)

Whole-cell lysates were lysed in NP-40 lysis buffer (Beyotime, P0013F) containing protease inhibitor cocktail (Beyotime, P1005) and PMSF (Beyotime, ST505). The cell lysates were centrifuged at 12,000 × g for 15 min, and the supernatants were collected and incubated with protein A/G magnetic beads (Vazyme, PB101-01) conjugated with specific antibodies. After overnight incubation, the magnetic beads were washed five times with IP washing buffer, and finally eluted with SDS-PAGE loading buffer for subsequent detection. Information of the antibodies used is as follows: p62 (Proteintech, 84,826–1-RR, 2 μg for 1 mg of total protein lysate), STAT3 (Proteintech, 10,253–2-AP, 2 μg for 1 mg of total protein lysate).

### Animal experiments

Four-week-old male ICR mice were purchased from the Experimental Animal Center of Nanjing University of Chinese Medicine and housed in a specific pathogen-free (SPF) environment. 3 × 10^5^ HepG2 cells were subcutaneously injected into the left axilla of the mice. After tumor formation, the mice were randomly divided into two groups with 5 mice in each group. Mice in the experimental group were intragastrically administered with 100 mg/mL Icaritin (Sigma-Aldrich, SML0551) once a week. All mice were sacrificed on day 28, and the tumors were removed. From day 7 onwards, the length and short diameter of the formed tumors were measured every 3 days. A growth curve was plotted based on the measured volume, and the tumor mass was weighed. This study was approved by Ethics Committee of China Pharmaceutical University (NO. 2024–12–072, Nanjing, China).

### Statistical analysis

Unpaired Student's *t* test was used to analyze differences between two groups. Data from three independent assays are presented as the mean ± SD. SPSS v22.0 software (IBM Corp.) was used for data analysis. One‑way ANOVA followed by LSD (one control group) or Tukey (more than one control group) post hoc test was used for comparing > 2 groups. *P* < 0.05 was considered to indicate a statistically significant difference.

## Results

### Icaritin diminishes HCC cell-mediated M2 polarization of macrophages by reprogramming metabolism

To uncover the efficacy of icartin on regulating HCC cell-mediated M2 polarization of macrophages. We firstly cocultured HCC cells and THP-1-derived macrophages in a Transwell system with 0.4 μm pores for 48 h to establish a coculture system in vitro (Fig. 1A). After cocultured with HCC cells, we found the secretion of IL-6 and TGF-β was increased in macrophages (Fig. 1B), and the expression of CD206, as the marker of M2 polarization, was also elevated (Fig. 1C). These results confirmed that HCC cells could induce macrophages polarize to M2 sub-populations. Subsequently, M0 macrophages were cocultured with HCC cells pretreated with or without icaritin (10uM) for 48 h (Fig. 1D). We found that secretion of IL-6 and TGF-β as well as CD206 expression in macrophages were decreased when cocultured with icaritin-pretreated HCC cells (Fig. 1E & F). To exclude the potential direct effect of residual icaritin on macrophages, we isolated TAMs after co-culture with HCC cells and treated them directly with or without icaritin. No significant changes were observed in the secretion of IL-6 and TGF-β (Fig. S1A) or in the mRNA expression of M2 markers compared to DMSO-treated controls (Fig. S1B). These results preliminarily demonstrate that icartin exerted the effect on blocking HCC cell-induced M2 polarization of macrophages.

To further determine whether icaritin inhibits M2 polarization by modulating HCC cell metabolism, THP-1-derived macrophages were exposed to the conditioned media (CM) harvested from HCC cells pretreated with or without icaritin (Fig. 1G). The results revealed that CD206 expression was decreased in macrophages administrated with the CM from icaritin-treated HCC cells (Fig. 1H). Given the critical role of metabolic reprogramming in macrophage polarization, we employed extracellular acidification rate (ECAR) and oxygen consumption rate (OCR) assay to assess metabolic pattens in macrophages. The results found that the OCR was increased in the macrophages with a reduction of ECAR after administrated with the CM from untreated HCC cells, but the effect was abolished by the CM from icaritin-pretreated HCC cells (Fig. 1I). Additionally, we detected the fatty acid oxidation (FAO) activity in macrophages and revealed that FAO activity was elevated after treated with HCC-derived CM, but this effect was also abrogated by CM from icaritin-pretreated HCC cells. (Fig. 1J). Collectively, these results demonstrate that icaritin diminished HCC cell-mediated metabolic reprogramming of macrophages, thereby imposing a functional blockade on M2 polarization.

### STX16 is identified as the novel potential target of icaritn in regulating HCC cells-mediated M2 polarization of macrophages

**Fig. 2:**
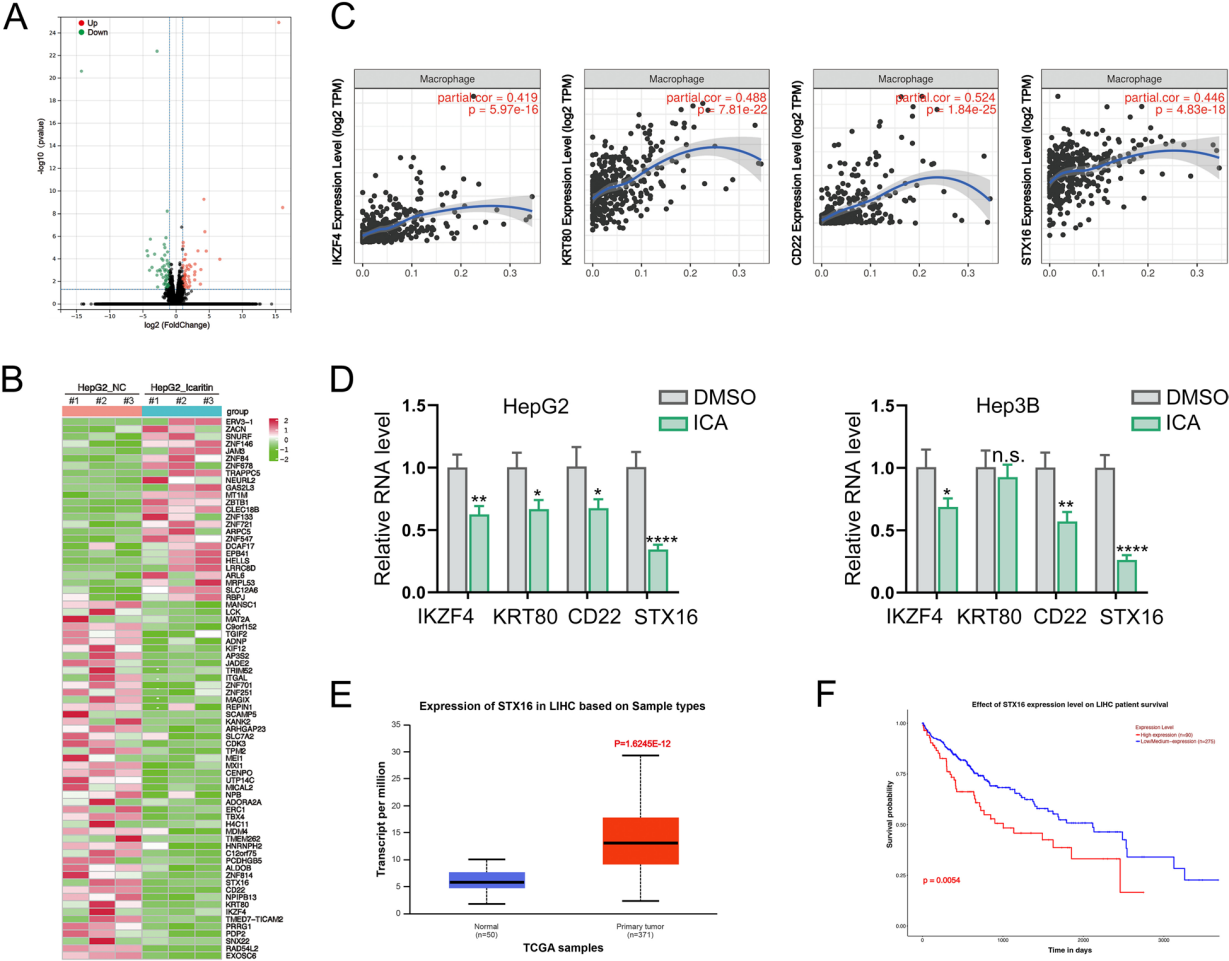
STX16 is identified as the novel potential target of icaritn in regulating HCC cells-mediated M2 polarization of macrophages. **A**. RNA-seq identification of differentially expressed genes in HCC cells treated with or without icaritin; **B**. Analysis of overexpressed and under expressed genes by heatmap; **C**. Identification of macrophages polarization related genes based on TIME database; **D**. Detection of candidate genes in HCC cells by qPCR; **E**. Analysis of STX16 expression in HCC tissues and adjacent tissues based on UALCAN database; **F**. Assessment of outcomes in HCC patients with high and low STX16 expression based on UALCAN database (*P< 0.05, **P< 0.01, ****P < 0.0001, n.s.: no significance)

To identify the underlying target of icaritin in HCC cells, RNA-seq assay was performed to analyze gene expression in HCC cells administrated with or without icaritin (Fig. 2A). mRNAs with differential expression (DEGs) listed in the NCBI database were filtered, yielding 25 upregulated and 49 downregulated genes (Fig. 2B). Next, we focused on downregulated genes associated with macrophage polarization, which were analyzed using TIMER with a correlation coefficient cutoff of > 0.4. IKZF4, KRT80, CD22, and STX16 were identified as potential targets (Fig. 2C). Given its most significant downregulation in icaritin-treated HCC cells, STX16 was selected as the target based on the results of qPCR assays (Fig. 2D). After analyzed the data from UALCAN database, we demonstrated that STX16 expressed highly in HCC tissues (Fig. 2E), and an increased expression of STX16 contributed to a poor prognosis (Fig. 2F). These findings indicate that STX16 may serve as a potential target for icaritin in inhibiting macrophage M2 polarization in HCC.

### STX16 is confirmed as the target of icaritin in regulating M2 polarization of macrophages in HCC

**Fig. 3:**
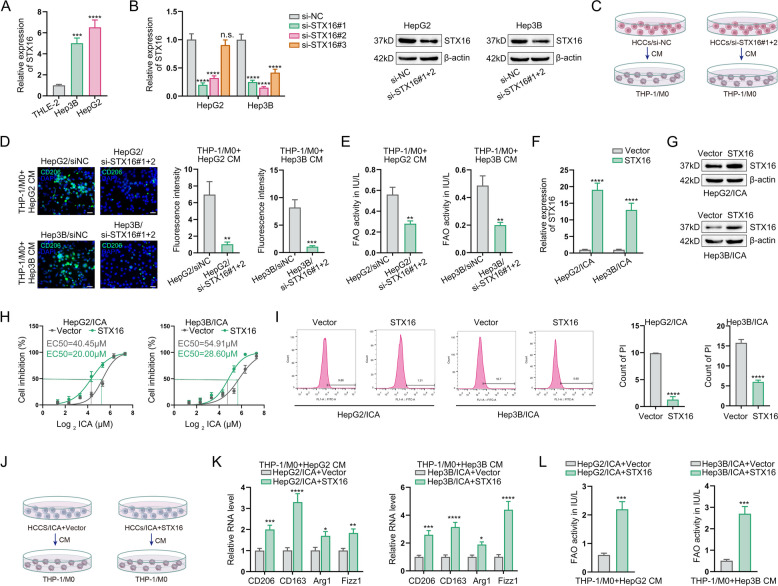
STX16 is confirmed as the target of icaritin in regulating M2 polarization of macrophages in HCC. **A** STX16 expression in HCC cells and normal hepatic cells was detected by qPCR; **B** Verification of si-STX16 interference efficiency in HCC cells by qPCR and western-blot assay; **C** Schematic of co-culturing macrophages with supernatants from STX16-silenced HCC cells; **D** IF detection of macrophage M2 polarization marker CD206; **E** Detection of FAO activity in macrophages after cocultured with STX16-silenced HCC cells; **F **& **G** Detection of STX16 overexpression efficiency by qPCR and western-blot assay after transfection; **H **& **I** CCK8 assay and PI flow cytometry detection of HCC cells sensitivity to icaritin after STX16 overexpression; **J** Schematic of macrophages treated with CM from icaritin-treated HCCs transfected with or without STX16 overexpression; **K** Detection of macrophage M2 polarization markers by qPCR; **L** Detection of FAO acclivity in macrophages (*P < 0.05, **P < 0.01, ***P < 0.001, ****P < 0.0001, n.s.: no significance)

To validate STX16 as a critical molecular determinant of macrophage polarization in HCC, we first performed comparative expression profiling across normal hepatocytes and HCC cell lines (Fig. 3A). High-expressing STX16 HCC lines were subjected to siRNA-mediated gene silencing, with qPCR and Western-blot confirming efficient knockdown (Fig. 3B). CM from STX16-silenced or NC-silenced HCC cells were then applied to macrophage cultures (Fig. 3C). Immunofluorescence analysis revealed that STX16 depletion in HCC cells significantly suppressed CD206 expression in co-cultured macrophages (Fig. 3D). Concomitantly, FAO activity was diminished in macrophages exposed to CM from STX16-silenced HCC (Fig. 3E). In a reciprocal gain-of-function approach, we transfected HCC cells with a pre-validated STX16 overexpression construct, confirming ectopic protein induction by qPCR and Western-blot (Fig. 3F-G). According to the results of CCK-8 and propidium iodide flow cytometry, it was demonstrated that STX16 overexpression rescued HCC cells from icaritin-induced cytotoxicity (Fig. 3H-I).

Next, we administrated macrophages with CM from icaritin pre-treated HCC cells with or without STX16 overexpression (Fig. 3J). qPCR results demonstrated that STX16 overexpression diminished the effect of icaritin on blocking HCC cells-induced M2 polarization of macrophages (Fig. 3K), concomitant with increased FAO activity (Fig. 3L). Collectively, these loss-of-function and gain-of-function experiments establish STX16 as a critical mediator through which icaritin suppresses HCC-induced M2 macrophage polarization.

### Icaritin-mediated STX16 downregulation blocks autophagosome-lysosome fusion in HCC cells, causing autophagosome accumulation and exocytosis via EVs

**Fig. 4:**
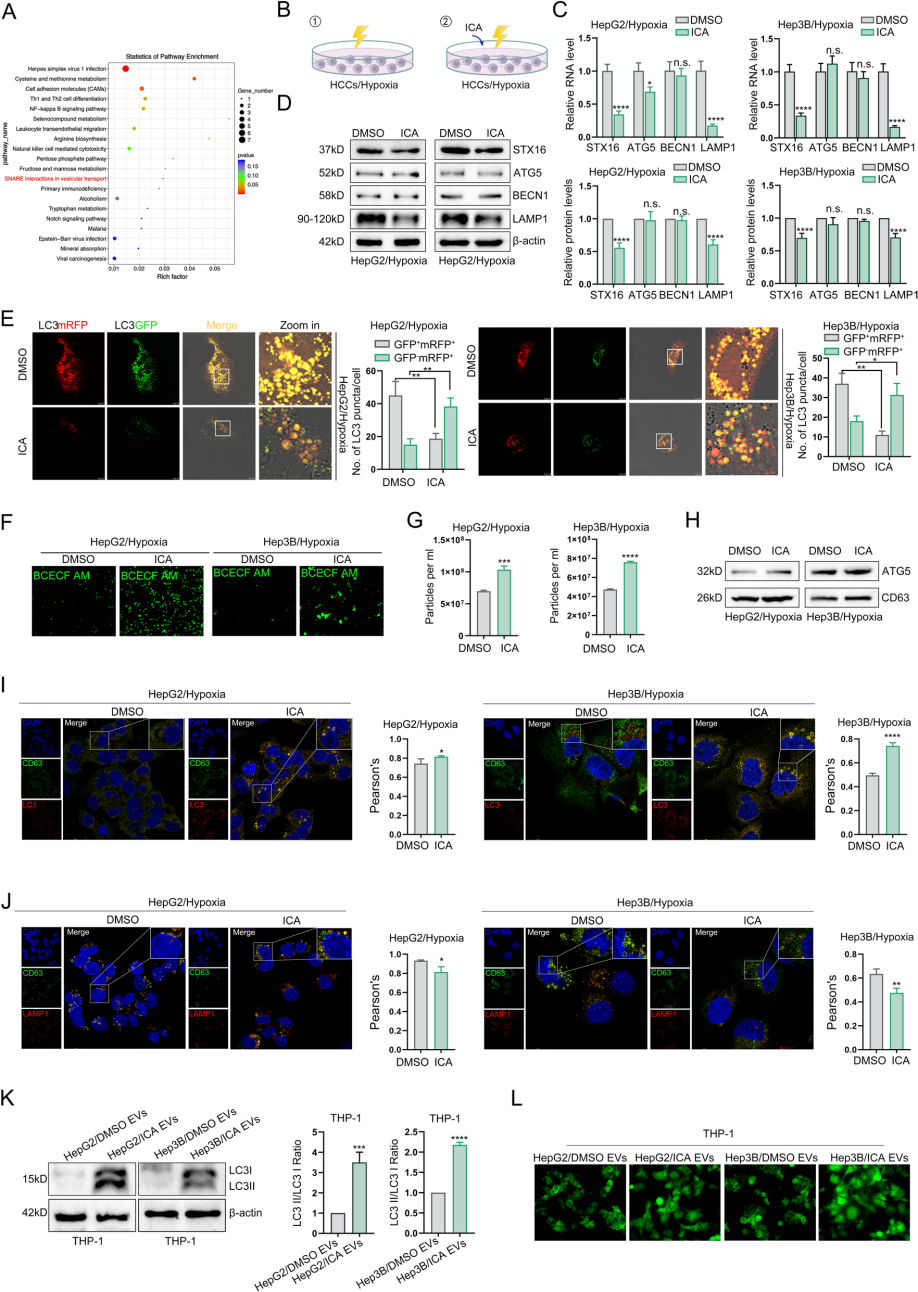
Icaritin downregulates STX16, blocking autophagosome-lysosome fusion in HCCs, causing autophagosome accumulation and exocytosis via EVs. **A** KEGG analysis of differentially expressed genes in HCC cells treated with or without icaritin; **B** Schematic of hypoxic autophagy-induced HCC cells treated with or without icaritin; **C **& **D** qPCR and western blot detecting for autophagy-related molecules; **E** Detection of autophagosome-lysosome fusion in HCC cells via mRFP-GFP-LC3 reporter system; **F** Detection of intracellular pH using BCECF-AM in HCC cells; **G** Detection of EVs content in HCC cells; **H** Analysis of autophagy in HCC cells-released EVs by western blot; **I** Immunofluorescence analysis showing co-localization EVs and the autophagosome in HCC cells treated with or without icaritin; **J** Immunofluorescence analysis showing co-localization of EVs and the lysosome in HCC cells treated with or without icaritin; **K** Western blot detection of LC3 in macrophages administrated by HCC cells-released EVs; **L** IF detection of autophagosome in macrophages after treated with HCC cells-released EVs (*P < 0.05, **P < 0.01, ***P < 0.001, ****P < 0.0001, n.s.: no significance)

KEGG pathway analysis of genes modulated by icaritin revealed significant enrichment of SNARE family members implicated in autophagosome-lysosome fusion (Fig. 4A), strongly suggesting that autophagic lysosome dysfunction underlies antitumor mechanism of icaritin. Given the critical role of autophagosome-lysosome fusion in autophagic flux, we inhibited lysosome biogenesis with CQ and BafA1, confirming impaired autophagolysosome formation via mRFP-GFP-LC3 puncta analysis (Fig. S2A). Functional assays revealed that blocking autophagic degradation in HCC cells abrogated their ability to promote M2 polarization, as evidenced by reduced CD206 expression, cytokine secretion, and FAO activity in co-cultured macrophages (Fig. S2B-E). These results demonstrate that disrupting autophagolysosome formation attenuates HCC-mediated M2 polarization of macrophages.

Previous investigations have established that syntaxin family members STX16 and STX17 are indispensable for autophagosome-lysosome fusion during autophagosome maturation [24, 25]. To dissect the role of STX16 in tumor cell autophagy, we modeled autophagic activation in the tumor microenvironment by exposing HCC cells to hypoxic conditions, with or without icaritin treatment (Fig. 4B). qPCR and Western blotting revealed that hypoxia upregulated autophagy-related genes (ATG5, BECN1) and fusion machinery components (STX16, LAMP1) in HCC cells (Fig. S2F-G). Conversely, icaritin treatment in hypoxic HCC cells selectively attenuated STX16 and LAMP1 expression, while leaving ATG5 and BECN1 expression unaltered (Fig. 4C-D).

Utilizing the mRFP-GFP-LC3 reporter system, we demonstrated that icaritin inhibited autophagolysosome biogenesis (Fig. 4E), which was accompanied by reduced intracellular acidity (Fig. 4F) and enhanced secretion of EVs encapsulating autophagosomes (Fig. 4G). Then, we examined the spatial relationship between the EVs marker CD63 and key autophagic-lysosomal compartments. In icaritin-treated HCC cells, we observed a significant increase in the co-localization of CD63 with LC3 (Fig. 4I), indicating an enhanced association of autophagosomes with EVs biogenesis or secretion machinery. Conversely, the co-localization between CD63 and the lysosomal marker LAMP1 was markedly reduced following icaritin treatment (Fig. 4J). Notably, EVs derived from icaritin-treated HCC cells induced autophagosome accumulation in macrophages, as evidenced by Western blot and fluorescence microscopy (Fig. 4K). These findings indicate that icaritin suppressed autophagy-lysosome fusion via STX16, leading to autophagosome accumulation in macrophages through EVs-mediated transportation.

### Icaritin inhibits histone lactylation-mediated STX16 transcriptionviaALDOB-dependent glycolysis in HCC

**Fig. 5:**
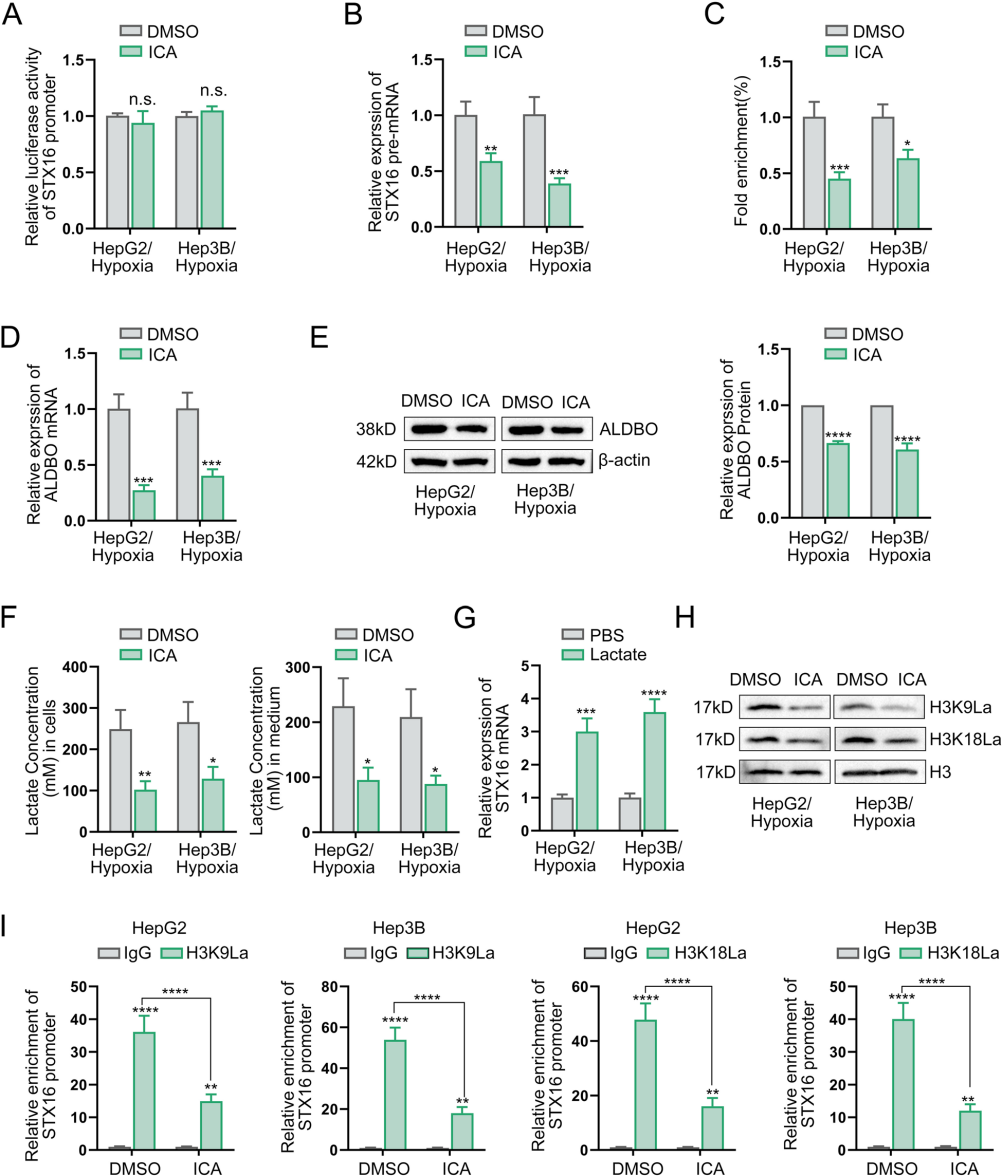
Icaritin inhibits histone lactylation-mediated STX16 transcription via ALDOB-dependent glycolysis in HCC. **A** Detection of STX16 promoter activity by dual-luciferase reporter assay in hypoxia-induced HCC cells treated with icaritin; **B** Detection of STX16 pre-mRNA expression in hypoxia-induced HCC cells administrated with or without icaritin; **C** ATAC-qPCR assay for detecting chromatin accessibility of STX16; **D **& **E** Examination of ALDOB expression in icaritin or DMSO treated HCC cells induced by hypoxia by qPCR and Western blot; **F** Detection of lactate concentration in HCC cells and their CM with DMSO or icaritin administration; **G** Examination of STX16 expression in hypoxia-induced HCC cells treated with or without lactate; **H** Detecting for modification of H3K9La and H318La in hypoxia-induced HCC cells administrated with or without icaritin; **I** ChIP-qPCR analysis of H3K9la and H3K18la modifications in the STX16 promoter in HCC cells with or without icaritin administration (*P < 0.05, **P < 0.01, ***P < 0.001, ****P < 0.0001)

To elucidate the molecular mechanisms underlying icaritin-mediated suppression of STX16, we first employed luciferase reporter assays to assess STX16 promoter activity. Notably, icaritin induced no significant alteration in promoter activity (Fig. 5A). Conversely, qPCR analysis showed that STX16 pre-mRNA levels were markedly reduced (Fig. 5B). This suppression of transcription was associated with a closure of chromatin, as evidenced by ATAC-qPCR assays which demonstrated that icaritin potently decreased chromatin openness at the STX16 locus (Fig. 5C). Integrative analysis of RNA-seq datasets revealed a significant decrease in ALDOB after icaritin treatment, pinpointing ALDOB, a glycolytic enzyme, as an additional target of icaritin. Given emerging evidence that lactate-driven histone lactylation promotes transcriptional activation [26], we postulated that icaritin-induced ALDOB downregulation reduces intracellular lactate levels, thereby impairing histone lactylation-dependent STX16 transcription. qPCR and Western blotting assay confirmed that ALDOB suppressed by icaritin in vitro (Fig. 5D-E). Concomitantly, lactate secretion assays revealed a markable reduction in extracellular lactate levels in icaritin-treated HCC cells (Fig. 5F). Notably, exogenous lactate supplementation rescued STX16 expression in icaritin-treated HCC cells (Fig. 5G). Histone H3 lysine 9 lactylation (H3K9La) and lysine 18 lactylation (H3K18La) have been reported to promote gene transcription. It has been detected that H3K9La and H3K18La modification levels had been reduced in icaritin-treated HCC cells (Fig. 5H). Concurrently, chromatin immunoprecipitation (ChIP)-qPCR assays focusing on the STX16 promoter region revealed diminished enrichment of both H3K9La and H3K18La modifications (Fig. 5I). These findings establish a mechanistic link whereby icaritin suppresses STX16 expression through ALDOB-mediated glycolytic repression and subsequent inhibition of histone lactylation (H3K9La/H3K18La) at the promoter site of STX16.

### Icaritin-induced autophagosome accumulation in HCC cells promotes autophagic cell death and inhibits M2 polarization in macrophages via EVs transfer

**Fig. 6:**
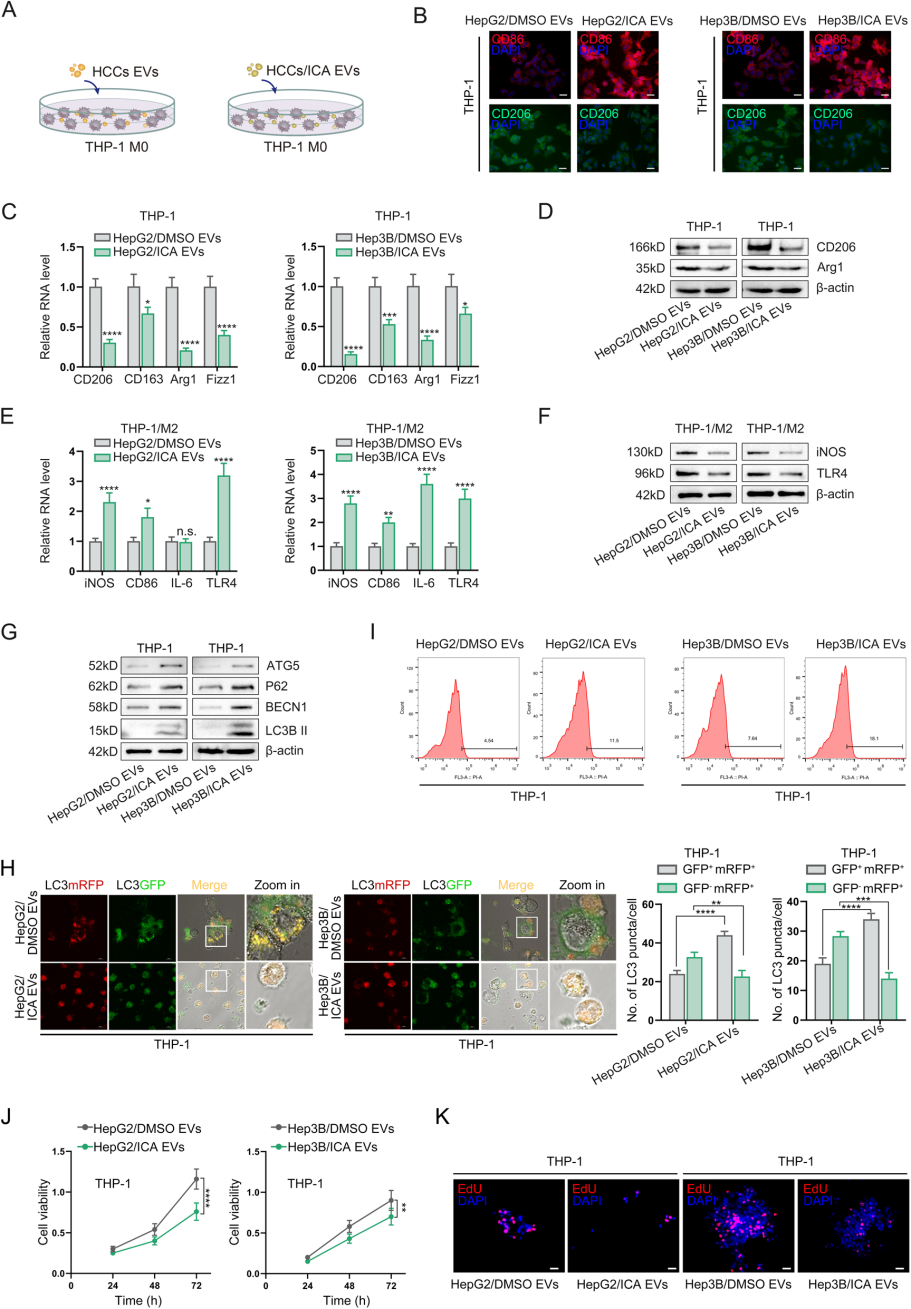
Icaritin-induced autophagosome accumulation in HCC cells promotes autophagic cell death and inhibits M2 polarization in macrophages via EVs transfer. **A** Schematic of macrophages treated with EVs derived from HCC cells; **B**, **C **& **D** IF, qPCR and Western blot detection of M2 polarization markers in macrophages with DMSO or icaritin induced EVs treatment; **E **& **F** qPCR and Western blot analysis of M1/M2 polarization markers in macrophage with different EVs treatment; **G**. Detection of autophagy-related genes expression in macrophages by Western-blot; H. Analysis of autophagolysosome biogenesis in macrophages using mRFP-GFP-LC3 reporter system; **I**. Flow cytometric analysis of ROS activity in macrophages with DMSO or icaritin induced EVs administration; **J **& **K**. Assessment of macrophages viability by CCK-8 assay and EdU stain (*P < 0.05, **P < 0.01, ***P < 0.001, ****P < 0.0001)

Considering EVs serve as key mediators of intercellular signaling in the tumor microenvironment [27], we isolated and characterized EVs from HCC cells with or without icaritin treatment (Fig. S3A-C). These EVs were then added to the culture supernatant of M0 macrophages to assess polarization state changes (Fig. 6A). Immunofluorescence, qPCR, and Western blot analyses revealed that EVs derived from icaritin-treated HCC cells significantly reduced the expression of M2 polarization markers in macrophages (Fig. 6B, C&D), while increased the levels of M1 polarization markers (Fig. 6E-F). Additionally, after administrated with icaritin-induced EVs, it was revealed that autophagy-related markers including ATG5, p62, BECN1, and LC3B-II, as well as labeled autophagolysosomes were increased in macrophages according to the results of western-blot and mRFP-GFP-LC3 reporter system (Fig. 6G & H). Lastly, icaritin-induced EVs treatment increased intracellular ROS levels in macrophages (Fig. 6I), while significantly reduced macrophages viability (Fig. 6J & K). Taken these considerations, it suggests that icaritin-induced EVs inhibit M2 polarization and cellular viability by transporting autophagosomes to macrophages.

### Autophagosome-loaded EVs from icaritin-treated HCC cells induce STAT3 degradation in macrophages to block M2 polarization

**Fig. 7:**
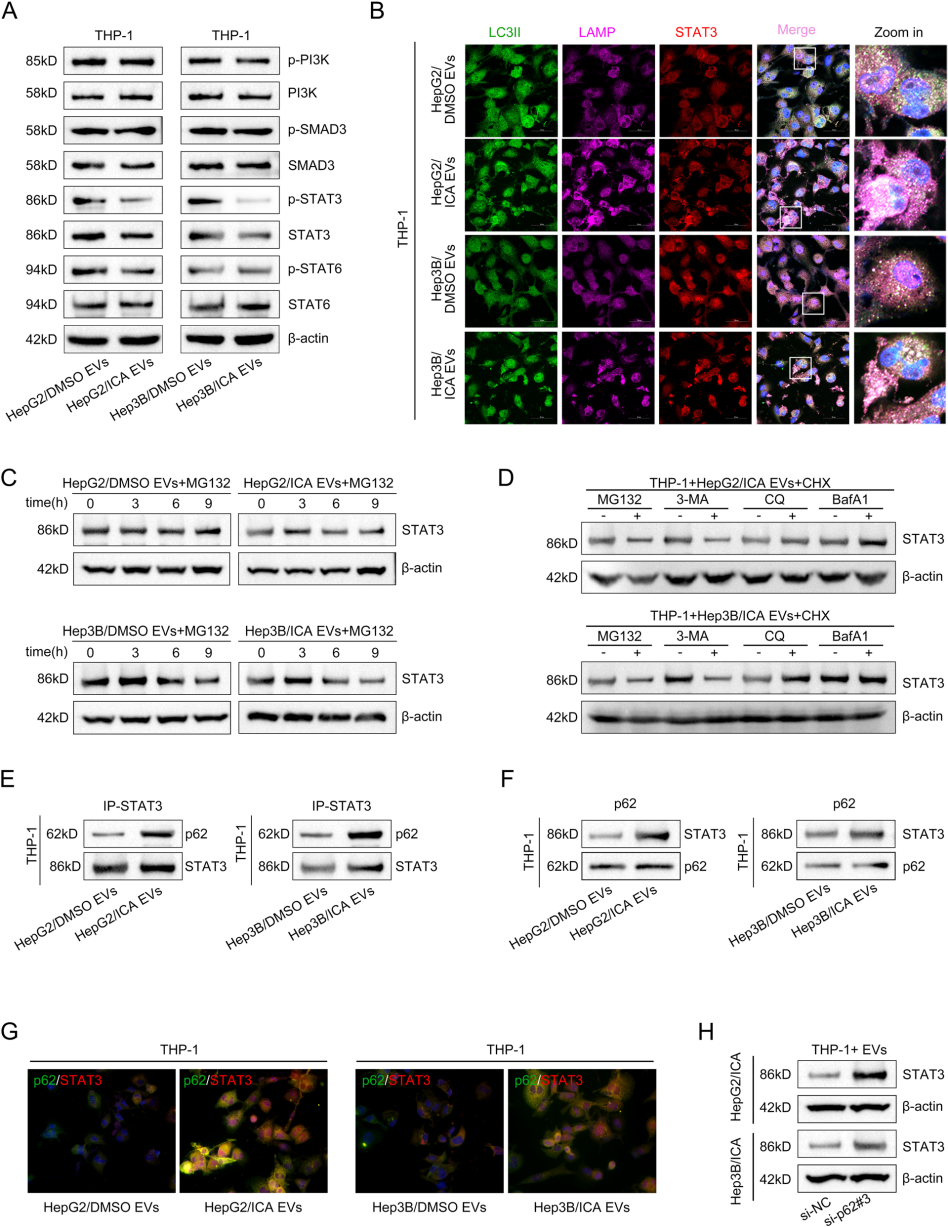
Autophagosome-loaded EVs from icaritin-treated HCC cells induce STAT3 degradation in macrophages to block M2 polarization. **A** Western-blot detection of protein expression and activity in macrophages treated with EVs; **B**. IF analysis of colocalization between STAT3 and autophagolysosomes in macrophages treated with icaritin or DMSO induced EVs; **C**. Analysis of STAT3 protein degradation level upon CHX treatment; **D**. Analysis of STAT3 degradation pathways in macrophages by administrated with proteasomal, autophagic, and lysosomal inhibitors; **E **& **F**. Co-IP assay for analyzing specific binding between STAT3 and p62 in EVs-treated macrophages; **G**. IF detection of subcellular colocalization between p62 and STAT3; **H**. Western blot detection of STAT3 expression in EVs-treated macrophages with or without p62-silenced

To identify the underlying target through which icaritin-induced HCC cell-derived EVs inhibit M2 polarization of macrophages, we detected the activity of PI3K, SMAD3, STAT3, and STAT6 by Western blot assay. It was revealed that icaritin-induced EVs significantly reduced both phosphorylated STAT3 (p-STAT3) and total STAT3 in M2-polarized macrophages (Fig. 7A), whereas STAT3 mRNA expression and stability remained unaffected (Fig. S4A & B). Immunofluorescence staining showed a marked increase in the colocalization of STAT3 with autophagolysosomes in macrophages treated with icaritin-induced EVs (Fig. 7B). Cycloheximide chase experiments further confirmed enhanced STAT3 degradation following EVs treatment (Fig. 7C). Then, we co-treated macrophages with icaritin-induced EVs and the proteasomal inhibitor MG132 or autophagy inhibitors (3-MA, CQ, BafA1). After detected by Western blot, we found that the lysosomal inhibitors CQ and BafA1, but not MG132 or 3-MA, abrogated STAT3 degradation (Fig. 7D), indicating that STAT3 degradation depended on the autophagolysosomal pathway upon treatment with icaritin-induced EVs.

To dig into how STAT3 gets degraded by EVs from icaritin-treated HCC cells, we firstly pulled down STAT3 from macrophages treated with either DMSO- or icaritin-induced EVs and detected its binding to p62 via Western blot. Turns out, icaritin-induced EVs boosted the interaction between STAT3 and p62 (Fig. 7E). Swapping the antibodies in reciprocal IP experiments confirmed these two proteins really bind specifically (Fig. 7F). Moreover, dual-fluorescence staining results showed they hang out together more in macrophages after icaritin-induced EVs treatment (Fig. 7G). Next, we knocked down p62 in macrophages (Fig. S4 C-D) and found that STAT3 levels bounced back (Fig. 7H), suggesting p62 drives autophagic degradation of STAT3. These findings demonstrated that icaritin-induced HCC cell-derived EVs inhibit macrophage M2 polarization by promoting STAT3 autophagic degradation via enhancing p62-STAT3 binding.

### Icartin inhibits tumor growth and HCC-mediated M2 polarization of macrophages in vivo

**Fig. 8:**
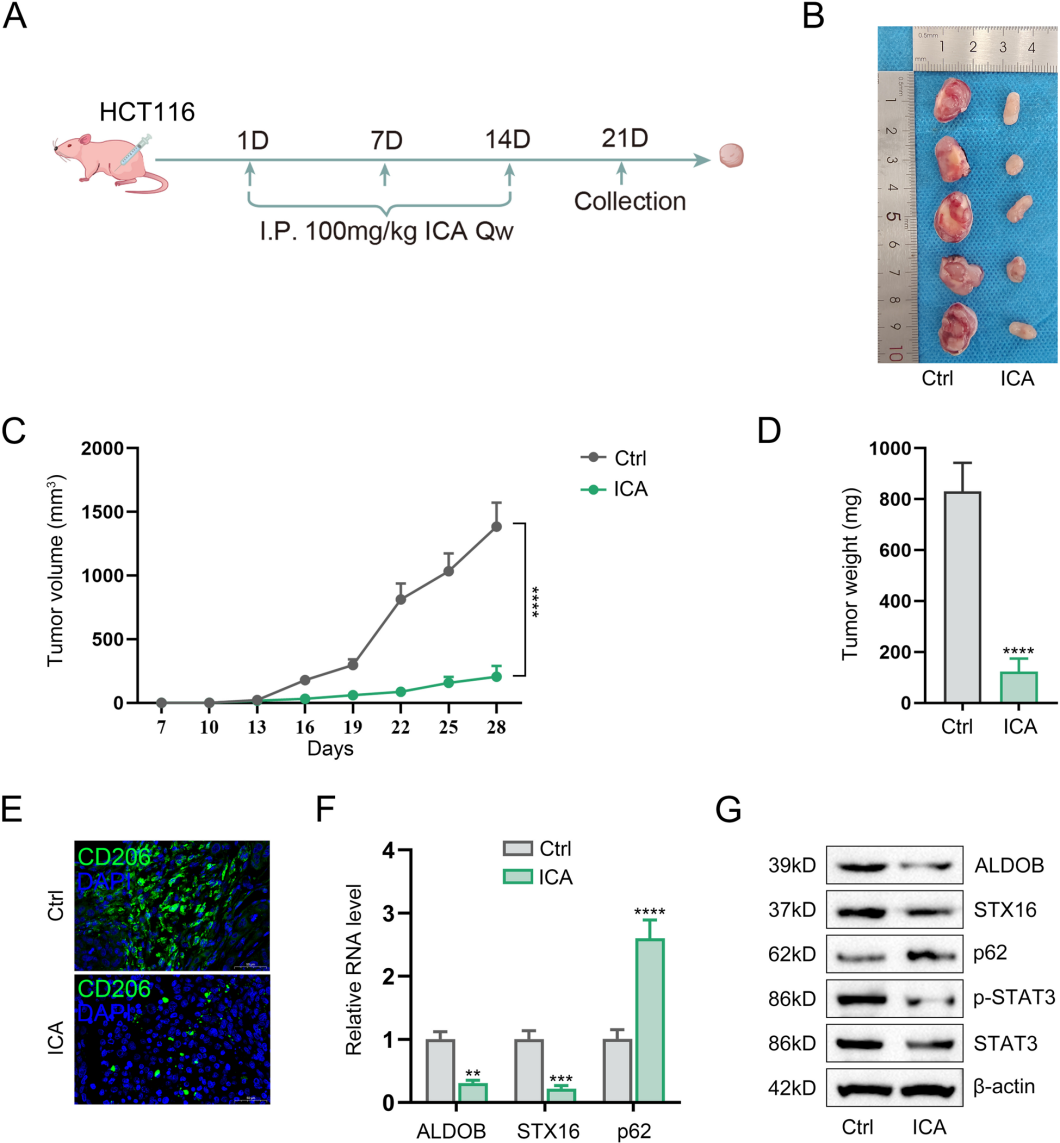
Icartin inhibits tumor growth and HCC-mediated M2 polarization of macrophages in vivo*.* **A** Schematic of tumor xenograft formation in nude mice; **B**. Tumor images on mice in each group treated with or without icaritin; **C**. Changes of tumor growth in each group; **D**. Tumor weight in each group; **E**. IF detection of CD206 expression in tumor tissues; **F**. Detection of ALDOB, STX16, and p62 expression in tumor tissues by qPCR; **G**. Examination of ALDOB, STX16, p62, STAT3, and pSTAT3 in tumor tissues by Western blot(**P < 0.01, ***P < 0.001, ****P < 0.0001)

To evaluate the in vivo antitumor efficacy of icaritin, HCC cells were subcutaneously injected into establish xenograft models (Fig. 8A). The mice were treated with icaritin or vehicle, and tumor length and width were measured every 3 days starting from day 7. Tumor tissues were harvested on day 21 (Fig. 8B) for growth curve plotting based on calculated volumes (Fig. 8C) and weight measurement (Fig. 8D). It was demonstrated that icaritin significantly suppressed tumor formation efficiency. IF staining results demonstrated that the expression of CD206 were decreased by icaritin administration (Fig. 8E). According to the results of qPCR (Fig. 8F) and Western blot (Fig. 8G), the expression of ALDOB, STX16, p62, STAT3, and p-STAT3 in tumor tissues were reduced after administrated with icaritin. These results confirmed that icaritin inhibits M2 polarization of macrophages via the ALDOB/STX16/autophagy/STAT3 pathway.

## Discussion

The liver orchestrates systemic metabolic homeostasis, making HCC uniquely consequential for metabolic dysregulation. Hepatocarcinogenesis drives profound metabolic reprogramming within tumor cells, which in turn remodels the immunosuppressive TME and directly compromises the efficacy of anticancer immunotherapies [28]. Accumulating evidence demonstrates that tumor-intrinsic metabolic reprogramming dictates the composition, polarization status, and effector functions of macrophages and other immune infiltrates [29, 30]. In present study, we demonstrated that icaritin abrogated M2 polarization of macrophages induced by co-cultured HCC cells. This effect of icaritin is associated with reprogramming the metabolic profiles of both HCC cells and macrophages. Specifically, icaritin inhibited glycolysis in hypoxia-induced HCC cells, while CM from icaritin-treated HCC cells suppressed FAO activity in macrophages. Previous study demonstrated that protumoral TAMs prefer to use mitochondria-dependent FAO as their energy supply [31]. Correspondingly, inhibition of FAO in TAMs impedes alternative polarization of TAMs toward protumor phenotype and inhibits tumor growth [32–34]. Additionally, our previous study established that glycolysis, as a core hallmark of cancer metabolism, derived HCC progression and could be effectively inhibited by icaritin [21]. These findings are consistent with the present study indicating that icaritin exerts an effect on reprogramming cancer metabolism, thereby modulating macrophage-mediated immunity in HCC. This study represents the first demonstration that icaritin regulates HCC cell glycolysis-mediated M2 macrophage polarization through a novel lactate-driven cascade.

Next, we identified ALDOB and STX16 as key targets through which icaritin abolishes macrophage M2 polarization mediated by metabolic reprogramming of HCC cells. Using gain- and loss-of-function approaches, we establish STX16 as an essential mediator of icaritin's suppression of HCC cell-driven M2 macrophage polarization. In addition, it was revealed that icaritin inhibited STX16 expression by targeting glycolysis mediated-histone lactylation in HCC cells. Mechanistically, icaritin downregulates the glycolytic enzyme ALDOB, reducing lactate production. This in turn inhibits H3K9la and H3K18la modifications, decreasing chromatin accessibility at the STX16 locus and suppressing its transcriptional activity. These findings uncover an insight perspective on TME remodeling through metabolic reprograming.

As the predominant glycolytic metabolite, lactate serves as a critical regulator of tumorigenic processes in HCC, including immunosuppressive microenvironment reprogramming [35]. Emerging evidence establishes lactate as an epigenetic regulator through histone lactylation—a recently identified post-translational modification that directly modulates transcription, gene expression, and cellular functions in neoplastic contexts [26]. Lactylation-mediated epigenetic reprogramming drives tumor progression and remodels the immunosuppressive TME [36–39]. Our findings establish a direct mechanistic link between metabolic reprogramming and histone lactylation, unveiling a novel paradigm for TME modulation in HCC therapy.

ALDOB catalyzes the cleavage of fructose-1,6-bisphosphate into dihydroxyacetone-3-phosphate and glyceraldehyde-3-phosphate, which plays a critical role in promoting glycolysis. However, mounting evidence suggests that ALDOB may also inhibit glycolysis through alternative mechanisms, such as binding to glucose-6-phosphate dehydrogenase or AKT in HCC [40, 41]. These findings indicate that ALDOB functions as a multifunctional molecule in the regulation of glycolytic reactions and HCC progression, with its functional duality potentially being dependent on the cells condition, such as hypoxia. Interestingly, tumor hypoxia constitutes a major barrier to T cell-mediated immunotherapy, as extensively reviewed for oxygen-supplied nanomaterials [42]. Our findings further underscore the necessity of overcoming hypoxia to rewire the immunosuppressive TME.

More interestingly, we uncovered a novel function of STX16 in HCC cell biology. As a member of the SNARE family, STX16 is critical for autophagosome-lysosome fusion. While both STX16 and STX17 belong to the SNARE family and participate in autophagic flux, they operate at different steps. STX17 is well-established as a key regulator of autophagosome-lysosome fusion by recruiting the HOPS complex and facilitating tethering. In contrast, STX16 primarily functions upstream by regulating lysosome biogenesis, acidification, and degradative capacity, thereby influencing autophagosome maturation [24]. Our data show that loss of STX16 did not block autophagosome formation but led to impaired intraluminal acidification and failed fusion with lysosomes, consistent with the expected role of STX16 in maintaining lysosomal activity. This distinction underscores the stage-specific vulnerability of the autophagic pathway to metabolic-epigenetic regulation in HCC.

Additional, Lai et al. developed a lysosome-targeted theranostic platform based on Zn(II)-Schiff base complexes, which integrates real-time fluorescence imaging with pH-responsive drug delivery for enhanced precision in cancer therapy [43], suggesting that lysosome-targeted therapeutic strategy is effective in cancer. Moreover, our data demonstrate that icaritin-induced STX16 downregulation reroutes autophagic cargo toward EV-mediated secretion. Supporting this model, we observed that icaritin treatment enhanced the spatial association between autophagosomes and EVs, while concurrently reducing the co-localization of EVs with lysosomes in donor HCC cells. This suggests a functional diversion of autophagosomes from the degradative lysosomal pathway to the secretory EV pathway upon fusion impairment, ultimately leading to their transfer to macrophages. Our study for the first time uncovers that EVs from HCC cells can shuttle autophagosomes and spark lethal autophagy in macrophages. Previous studies demonstrated that active autophagy led to M1 polarization of macrophages [44, 45], which consist with current study. By using fluorescent labeling and co-culture systems, we visualized the transfer of autophagosomes from HCC-derived EVs to macrophages, which was accompanied by increased autophagic flux and ROS production in macrophages, leading to autophagic cell death. Additionally, EVs released from icaritin-treated HCC cells activate the autophagic degradation of STAT3 upon uptake by macrophages, blocking key molecule for M2 polarization [46, 47]. Our work paves the way for innovative strategies to eliminate M2-like TAMs by targeting EVs-mediated autophagic cargo transport.

Despite elucidating the multiple mechanisms by which icaritin regulates macrophage polarization, several questions remain. Firstly, the specific molecular recognition mechanism of EVs-mediated autophagosome transfer is unclear, such as how autophagosomes are selectively loaded into EVs and whether this process depends on specific membrane proteins (e.g., STX16). Moreover, the effects of icaritin on other immune cells (e.g., T cells, neutrophils) in the TME are uninvestigated, which may influence the evaluation of its overall antitumor immune effect. Besides, our co-localization data functionally support the rerouting of autophagosomes into the EVs pathway, the definitive ultrastructural visualization of these cargoes within EVs, via techniques such as immunogold TEM, remains a subject for future investigation. Finally, this study lacks correlation analysis of the ALDOB/STX16/STAT3 pathway in clinical samples, requiring further validation using HCC patient cohorts in future research. Future studies could focus on proteomic analysis of EVs-carried autophagosomes to identify key cargo proteins and regulatory factors, while exploring the combinatorial potential of icaritin with ICIs to provide a theoretical basis for precision immunotherapy in HCC.

## Supplementary Information

Supplementary Material 1.

## Data Availability

The original contributions presented in the study are included in the article/supplementary material. Further inquiries can be directed to the corresponding authors.
