# Supplementary material for: Icaritin eliminates tumor-associated macrophages via STX16-dependent extracellular vesicle delivery of autophagosomes from hepatocellular carcinoma cells
Source: J Exp Clin Cancer Res. 2026 Feb 23;45:80. doi: 10.1186/s13046-026-03671-0 (PMC13032526; doi:10.1186/s13046-026-03671-0)
Supplement: Supplementary file 1 — Supplementary Material 1. [file 13046_2026_3671_MOESM1_ESM.docx]

Table S1. The primers sequence of transfected gene.

| Gene | Forward Primer（5' -3'） | Reverse Primer（5' -3'） |
| --- | --- | --- |
| si-NC | UUCUCCGAACGUGUCACGUUU | ACGUGACACGUUCGGAGAAUU |
| si-STX16 #1 | GGAACAUGCCAUUGAGAUAUU | UAUCUCAAUGGCAUGUUCCUU |
| si-STX16 #2 | CAUGAAGAAUCGAGAGGAAUU | UUCCUCUCGAUUCUUCAUGUU |
| si-STX16 #3 | GUACAGUCCUUGACAGAAUUU | AUUCUGUCAAGGACUGUACUU |
| si- p62#1 | CAGCAAGCCGGGUGGGAAUUU | AUUCCCACCCGGCUUGCUGUU |
| si- p62#2 | GGAAAUGGGUCCACCAGGAUU | UCCUGGUGGACCCAUUUCCUU |
| si-p62#3 | GGAUAACUGUUCAGGAGGAUU | UCCUCCUGAACAGUUAUCCUU |
| pcDNA3.1-STX16 | cggaattcATGGCCACCAGGCG | cgggatccTTATCGAGACTTCACGCC |

Table S2. Primer sequences of related genes.

| Gene | Forward primer (5’-3’) | Reverse primer (5’-3’) |
| --- | --- | --- |
| IKZF4 | TGATGCACTCACTGGTCACC | CTCCTCCAGGGTACTCTGCT |
| Fizz1 | GACTGCTACTGGGTGTGCTT | TTAGGACAGTTGGCAGCAGC |
| CD163 | TCCTTGTGGGATTGTCCTGC | AAGCCGCTGTCTCTGTCTTC |
| CD86 | ACACGGTTACCCAGAACCTA | CGCGTCTTGTCAGTTTCCAG |
| iNOS | GAGGAGATGCTGGAGATGGC | AAACATAGAGGTGGCCTGGC |
| CD206 | AGGATGGGTACTGGGCAGAT | TCCTGCAGCCTTTTTCGACT |
| Arg1 | AAGATTCCCGATGTGCCAGG | GGTCCAGTCCGTCAACATCA |
| STX16 pre-mRNA | TGTTGGCCGGATTAAGCAGA | TGAGAAGGCTCGCTGGTTTC |
| KRT80 | AGGAGGTGAAGGCCCAGTAT | GATGCGCACATTGAGATCCG |
| CD22 | GAAACTCGGTCAGCCTCCAA | CACTTCAAGTGTCCAGGCCT |
| STX16 | AGTCAGGCTACCTCAAACGC | ATCATCGCCCCTAAGTCCCT |
| ATG5 | TGGGCCATCAATCGGAAACT | TGTGCAACTGTCCATCTGCA |
| BECN1 | ATAACTTCAGGCTGGGTCGC | GTCCCAGAAAAACCGCAACC |
| LAMP1 | CACACCTTTTCCCCAATGCG | AAAGGTACGCCTGGATGGTG |
| ALDBO | CACCCAGGAGCAGAAGAAGG | CCACCTTGATCCTCTGCAGG |
| TLR4 | AAAATCCCCGACAACCTCCC | AAAGGCTCCCAGGGCTAAAC |
| IL-6 | TGAGGAGACTTGCCTGGTGA | GGTCAGGGGTGGTTATTGCA |
| P62 | ACAGGTGAACTCCAGTCCCT | AGATGTGGGTACAAGGCAGC |
| ACTB | AGAAAATCTGGCACCACACC | CCATCTCTTGCTCGAAGTCC |
| STX16 | TGGATGACAGCAGCGAAGAG | CTGGTGGAGAGTTCCTGCAG |
| STAT3 | CAGTTTCTGGCCCCTTGGAT | TGATTCTTCCCACAGGCACC |
| ALDBO（M） | AACCGTTGCCCTCTACCAAG | TGAACGTACTGTCCTTGGGC |
| STX16（M） | CGCTTTCTTGTTGTTGCGGA | TGCGGCCAACATCATACTGT |
| P62（M） | TGGGGACTTGGTTGCCTTTT | ATCACATTGGGGTGCACCAT |
| ACTB（M） | GCCATGTACGTAGCCATCCA | TCACGCACGATTTCCCTCTC |

**Table S3. Primary antibodies used for Western blot analysis**

| **Target** | **Vendor (Catalog #)** | **Dilution** |
| --- | --- | --- |
| β-actin | Proteintech (66009-1-Ig) | 1:100,000 |
| STX16 | Proteintech (11201-1-AP) | 1:2,000 |
| ATG5 | Proteintech (10181-2-AP) | 1:5,000 |
| BECN1 | Proteintech (11306-1-AP) | 1:5,000 |
| LAMP1 | Proteintech (67300-1-Ig) | 1:10,000 |
| LC3B | CST (2775S) / Proteintech (14600-1-AP) | 1:1,000 / 1:5,000 |
| p62/SQSTM1 | Proteintech (84826-1-RR) | 1:10,000 |
| ALDOB | Proteintech (18065-1-AP) | 1:10,000 |
| H3K18la | PTM Bio (PTM-1406RM) | 1:2,000 |
| H3K9la | PTM Bio (PTM-1419RM) | 1:2,000 |
| STAT3/p-STAT3 | Proteintech (10253-2-AP/60479-1-Ig) | 1:10,000 |
| CD206 | CST (24595S) | 1:1,000 |
| Arg1 | Proteintech (16001-1-AP) | 1:1,000 |
| Calnexin | Proteintech (10427-2-AP) | 1:10,000 |


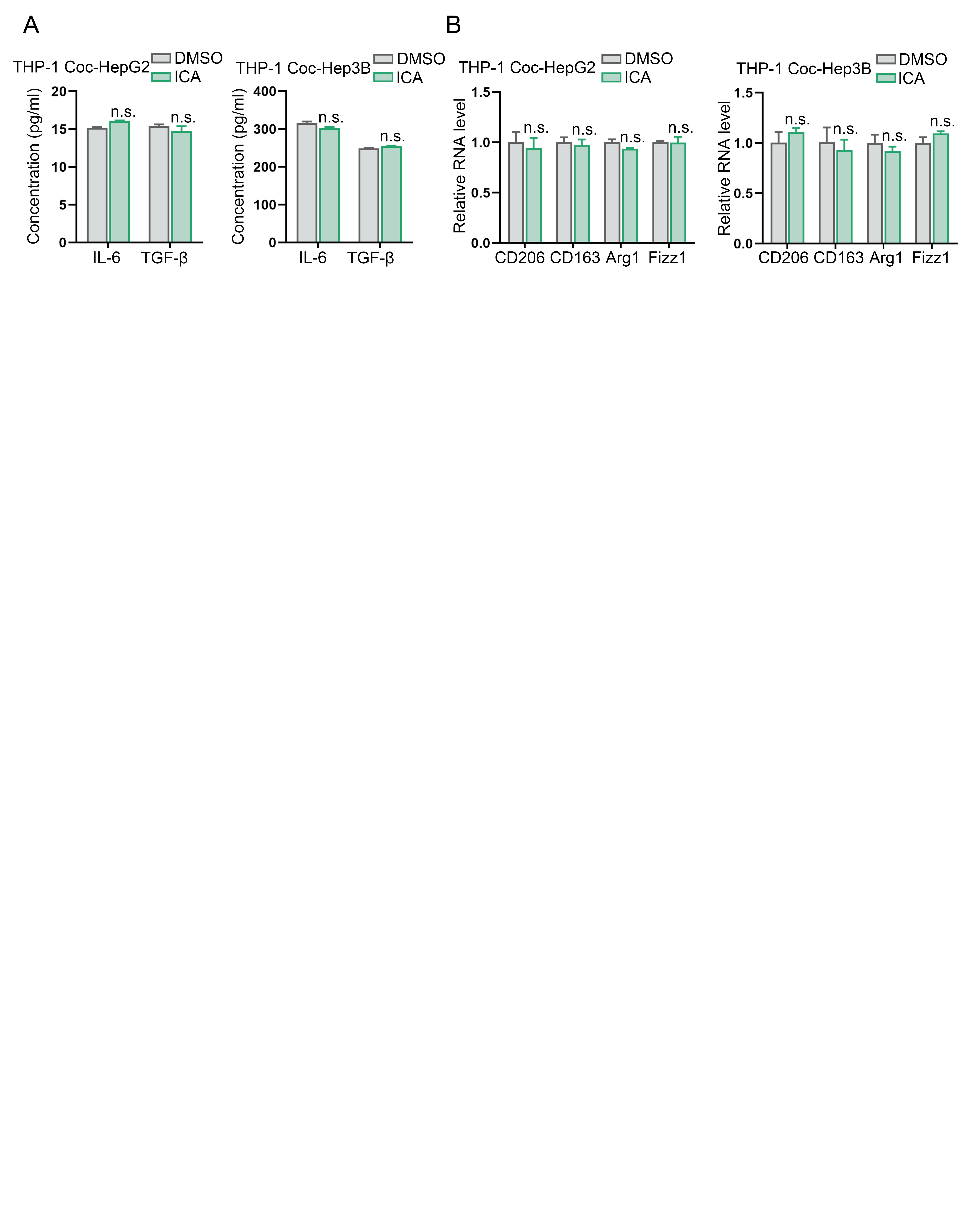


**Figure S1. Direct treatment with icaritin does not affect the phenotype of pre-polarized tumor- TAMs.**

A. Secretion levels of IL-6 and TGF-β in the supernatant were measured by ELISA; B. mRNA expression levels of classical M2 markers (CD206, CD163, Arg1, Fizz1) were analyzed by qRT-PCR.


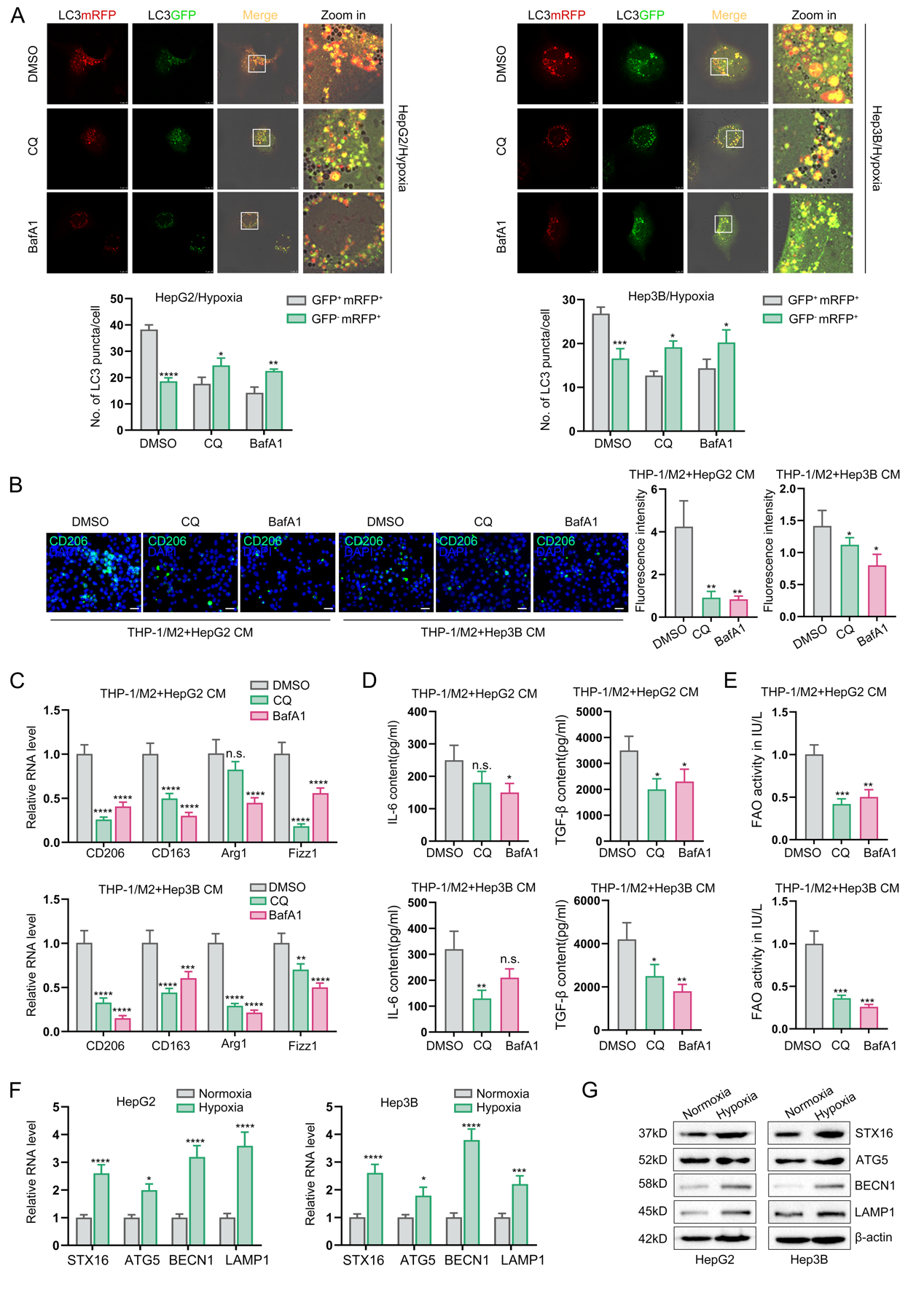


**Figure S2. Autophagy involves in affecting HCC cells-mediated M2 polarization of macrophages.**

A. Analysis of autophagolysosome biogenesis in macrophages treated with CQ or BafA1 using mRFP-GFP-LC3 reporter system; B&C. IF and qPCR detection of polarization markers expression in macrophages cocultured with CM from HCC cells pre-treated with CQ or BafA1; D. ELISA examination of IL-6 and TGF-β secretion in macrophages cocultured with CM from HCC cells pre-treated with CQ or BafA1; E. Detection of FAO activity in macrophages cocultured with CM from HCC cells pre-treated with CQ or BafA1; F&G. Detection of autophagy-related genes in hapoxia-induced HCC cells by qPCR and Western blot.


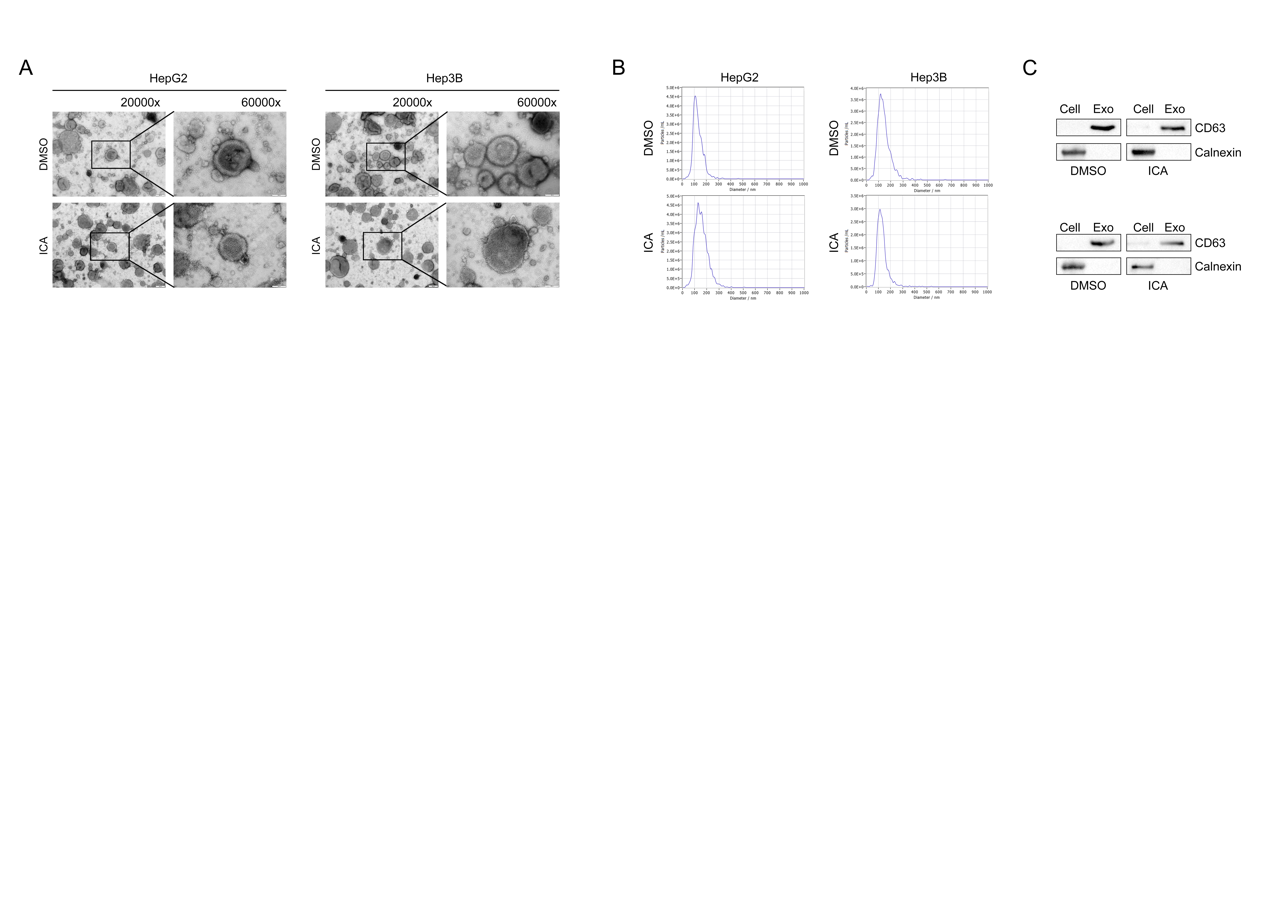


**Figure S3. Identification of exosomes delivered from HCC cells.**

A. Electron microscopy detection of isolated exosomes; B.NTA analysis of particle size and concentration of isolated exosomes; C. Detection of exosome markers by Western blot.


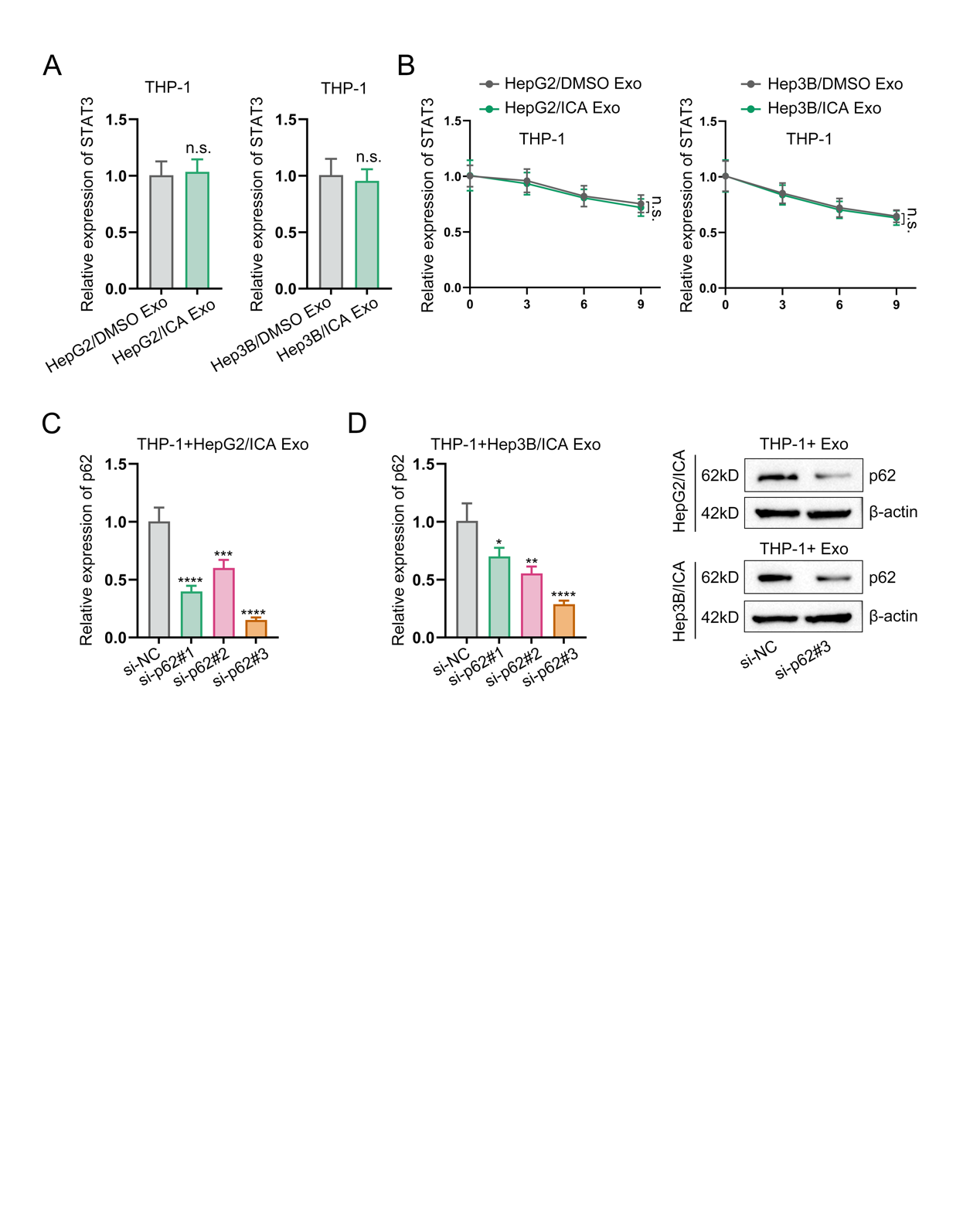


**Figure S4.** A. Detection of STAT3 expression in macrophages pretreated with HCC cells-isolated exosomes; B. analysis of STAT3 mRNA stability in macrophages pretreated with HCC cells-isolated exosomes; C&D. verification of salience quality of p62 in macrophages by qPCR and Western blot.
